# Electrochemotherapy as a new therapeutic strategy in advanced Merkel cell carcinoma of head and neck region

**DOI:** 10.2478/raon-2013-0059

**Published:** 2013-10-08

**Authors:** Daniele Scelsi, Niccolò Mevio, Giulia Bertino, Antonio Occhini, Valeria Brazzelli, Patrizia Morbini, Marco Benazzo

**Affiliations:** 1 Department of Otolaryngology Head Neck Surgery, University of Pavia, IRCCS Policlinico S. Matteo Foundation, Pavia, Italy; 2 Institute of Dermatology, University of Pavia, IRCCS Policlinico S. Matteo Foundation, Pavia, Italy; 3 Department of Molecular Medicine, Section of Pathology, University of Pavia, IRCCS Policlinico S. Matteo Foundation, Pavia, Italy

**Keywords:** electrochemotherapy, head and neck cancer, Merkel cell carcinoma, palliative treatment

## Abstract

**Background:**

Merkel Cell Carcinoma (MCC) is a rare and aggressive tumour, arising from a cutaneous mechanoceptor cell located in the basal layer of epidermis, with poor prognosis. The treatment of choice for the initial stage of the disease is surgery and/or radiotherapy. The treatment of recurrent or advanced disease is still controversial.

**Case report:**

We report a case of 84 years old woman with a recurrent MCC of the chin treated with electrochemotherapy (ECT). During the period of 20 months, four sessions of ECT were employed, which resulted in an objective response of the tumour and good quality of residual life.

**Conclusions:**

Our case shows the effectiveness of ECT in the treatment of locally advanced MCC of the head and neck region in a patient not suitable for standard therapeutic options.

## Introduction

Merkel Cell Carcinoma (MCC) is a rare and aggressive cancer described for the first time in 1972 by Toker *et al.*^1^ Clinically it is characterized by a rapidly increasing red or bluish nodule. It frequently occurs in the head and neck area (41–50%), followed by upper and lower limbs (32–38%) and trunk (12–14%). This neuroendocrine neoplasia arises from a cutaneous mechanoceptor cell (Merkel cell), located in the basal layer of epidermis.^1^ The annual incidence in the Caucasian population is 0.23 per 100,000 individuals and 61% of these cases are men. MCC affects more frequently elderly and immunecompromised patients with a previously history of damaging sun exposure.^2^ The prognosis is poor due to the fast local growth and high local recurrence, regional lymph node metastases and distant metastases rates, occurring even after the prompt treatment.^3^

The main treatment modality for the early stage tumours is surgery and/or radiotherapy, while chemotherapy with etoposide and cisplatin or carboplatin finds a role only in patients with systemic metastases. Due to the low incidence of MCC, the experiences with recurrent lesions or advanced stage tumours are scarce.^3^,^4^

We report the case of a 84 years old woman affected by a extensive recurrence of MCC of the chin treated with multiple sessions of electrochemotherapy (ECT).

## Case report

**FIGURE 1.**
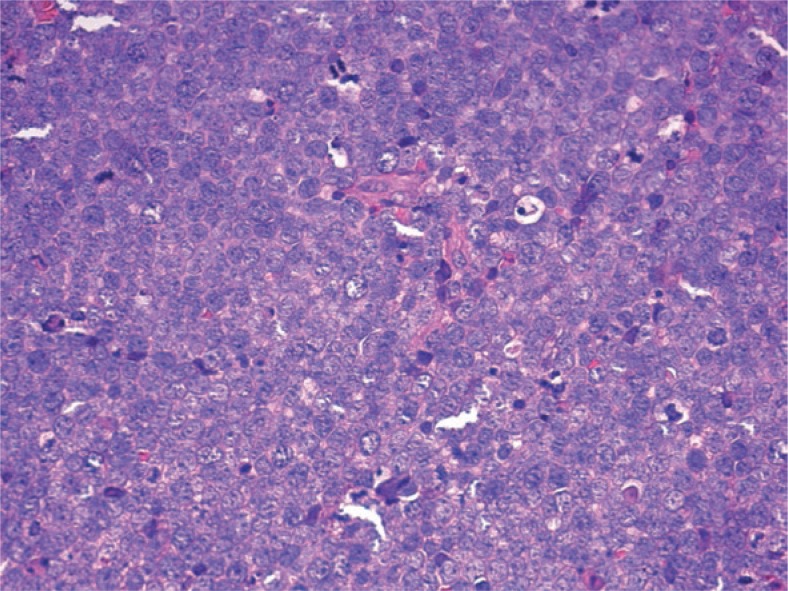
Tumour biopsy showing poorly differentiated cells with scarce cytoplasm and vesicular nuclei with inconspicuous nucleoli. High mitotic index and apoptotic figures were present. Immunohistochemical reactions were positive for cytokeratin 8 and 20 and neuroendocrine markers (chromogranin, synaptophysin and CD56/NCAM); TTF-1 was not expressed (H&E stain, 40×).

A 84 years old woman with hypertension, chronic vascular disease, chronic cardiac ischemia was admitted because of a dome-shaped bluish skin lesion in her chin of 2x2 cm, characterized by rapid growth. A skin biopsy showed poorly differentiated cells with scarce cytoplasm and vesicular nuclei with inconspicuous nucleoli. High mitotic index and apoptotic figures were present. Immunohistochemical reactions were positive for cytokeratin 8 and 20 and neuroendocrine markers chromogranin, synaptophysin and CD56/N-CAM; TTF-1 was not expressed. A diagnosis of MCC was established (Figure 1).

Total body computed tomography scan and bone scintigraphy excluded mandibular infiltration, regional or systemic metastases. The lesion was staged cT2N0M0. The patient underwent surgical excision of the tumour, bilateral selective neck dissection (levels I–III) and reconstruction with radial forearm free flap. The histopathological examination of resected specimen confirmed a stage IIA (pT2N0M0) MCC and surgical margins free of tumour. The patient refused proposed postoperative radiotherapy, whereas the systemic therapy was not indicated due to co-morbidities.

**FIGURE 2.**
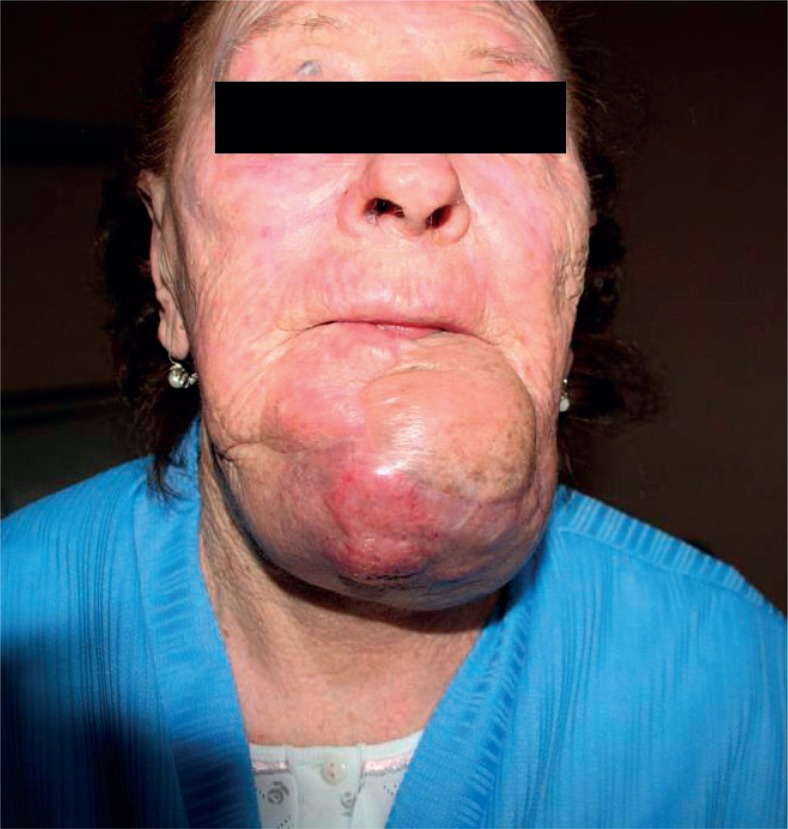
Before first electrochemotherapy: voluminous bluish lesion of the chin.

Two months after the treatment, mass in the area of previous surgery extending into a submandibular region was documented. A biopsy of the mass confirmed recurrent MCC (Figure 2). The re-operation was not an option because of the tumour extension, and radiotherapy was again refused by the patient. She was offered a palliative treatment with ECT. The treatment was performed according to the standard operating procedures of the ESOPE.^5^ Intravenous bleomycin infusion of 15.000 IU/m^2^ was administered eight minutes before delivery of electric pulses under general anaesthesia by means of a hexagonal electrode (length 25 mm) connected to a generator (Cliniporator^™^ - IGEA srl, Carpi, [MO], Italy). The lesion with 1 cm tumour-free margin was treated by multiple direct insertions of the electrode. The pulses were completed within 28 minutes after the infusion of bleomycin.

**FIGURE 3.**
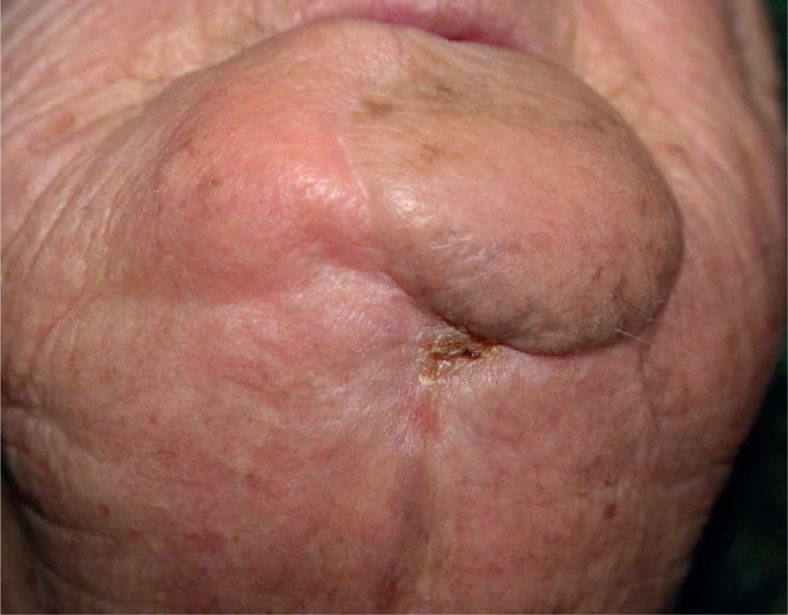
Reduction of 80% of the volume to the initial lesion after 2 treatments.

The patient was evaluated four weeks after the treatment: 50% reduction of the original tumour volume was observed, corresponding to a partial response according to RECIST criteria.^6^ The next ECT treatment resulted in 80% volume reduction compared to the original lesion (Figure 3).

**FIGURE 4.**
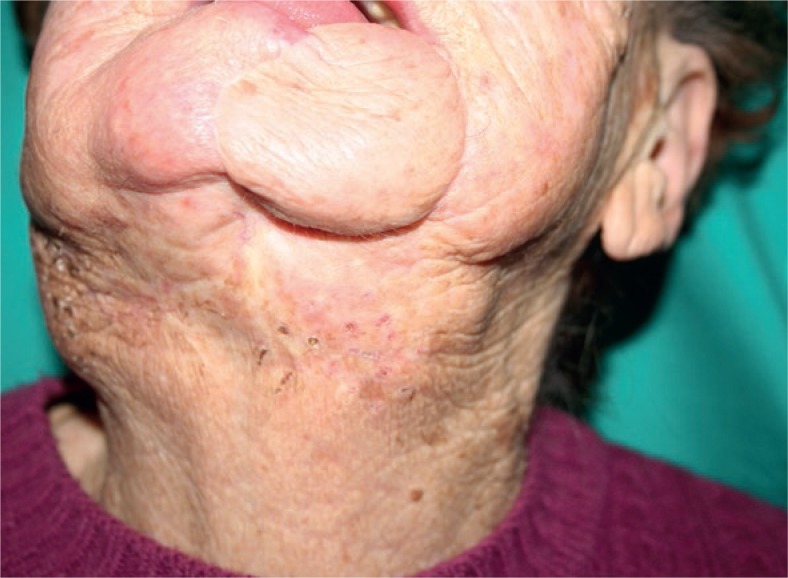
Follow up after four electrochemotherapy applications and 16 months.

Due to the good response and tolerability of the procedure, two additional ECT treatments were performed during the following sixteen months, which resulted in a good local control of the tumour (Figure 4).

Unfortunately, just after the fourth ECT treatment (and 20 months after the first ECT session), lung metastases were diagnosed. The patient died one month later due to heart failure which was unrelated to MCC.

No major complications were observed during or after ECT treatments. The only recorded side effect was moderate pain after the first ECT session, easily managed with peroral non-opioids. The patient reported a good quality of life until the last two months before she died when severe pain, controlled by opioids, appeared.

## Discussion

The management of patients with MCC of the head and neck region is still challenging due to poor prognosis. According to the National Comprehensive Cancer Network guidelines^7^, surgery is commonly considered the treatment of choice and significantly improves the overall survival, if associated with adjuvant radiotherapy.^8^ In the head and neck area where it is difficult to obtain safety margins wide enough, radiotherapy can be the first treatment option.^3^ Because of the high incidence of occult regional metastasis, patients with clinical and radioghaphically negative necks should undergo elective dissection, irradiation, or preferably sentinel lymph node biopsy. Chemotherapy is an option for patients with an incurable recurrent, heavily pre-treated disease or for those with systemic metastases although without proven effect on the overall survival.^9^

In the last years, ECT has been proposed as a novel therapeutic weapon for the control of recurrent cutaneous, subcutaneous or mucosal neoplastic lesions of different histologies.^5^ In the literature, few data exist about the effectiveness of this procedure in the treatment of head and neck cancers; the reported rates of objective response (OR) seem promising, ranging from 56% to 100%, depending on the tumour size.^10^–^16^ To the best of our knowledge there is only one case of MCC treated with ECT documented in the literature. The authors reported a complete response (CR) of the tumour to ECT after a follow up time of six months.^12^

The choice of using ECT in our patient was determined by the existing comorbidities and the patient’s refusal of radiotherapy. With ECT we were able to reach a good local tumour control, with no significant adverse events, nor functional or esthetic, which resulted in a good quality of the rest of her life. This case demonstrates that ECT can be considered as an effective palliative treatment option for patients with recurrent or advanced-stage tumour, not suitable for conventional treatments.^10^–^14^
